# Identification of mitochondrial RNA polymerase as a potential therapeutic target of osteosarcoma

**DOI:** 10.1038/s41420-021-00780-x

**Published:** 2021-12-14

**Authors:** Qi-cai Han, Xiang-yang Zhang, Peng-hui Yan, Song-feng Chen, Fei-fei Liu, Yun-Rong Zhu, Qing Tian

**Affiliations:** 1 Department of Orthopaedics, The First Affiliated Hospital of Zhengzhou University, 450052 Zhengzhou, China; 2 Department of Orthopaedics, Tongren Hospital, Shanghai Jiao Tong University School of Medicine, Shanghai, China; 3 Department of Orthopedics, Affiliated Jiangyin Hospital of Medical College of Southeast University, Jiangyin, China

**Keywords:** Bone cancer, Oncogenes

## Abstract

POLRMT (RNA polymerase mitochondrial) is essential for transcription of mitochondrial genome encoding components of oxidative phosphorylation process. The current study tested POLRMT expression and its potential function in osteosarcoma (OS). The Cancer Genome Atlas (TCGA) cohorts and Gene Expression Profiling Interactive Analysis (GEPIA) database both show that *POLRMT* transcripts are elevated in OS tissues. In addition, *POLRMT* mRNA and protein levels were upregulated in local OS tissues as well as in established and primary human OS cells. In different OS cells, shRNA-induced stable knockdown of POLRMT decreased cell viability, proliferation, migration, and invasion, whiling inducing apoptosis activation. CRISPR/Cas9-induced POLRMT knockout induced potent anti-OS cell activity as well. Conversely, in primary OS cells ectopic POLRMT overexpression accelerated cell proliferation and migration. In vivo, intratumoral injection of adeno-associated virus-packed POLRMT shRNA potently inhibited U2OS xenograft growth in nude mice. Importantly, levels of mitochondrial DNA, mitochondrial transcripts and expression of respiratory chain complex subunits were significantly decreased in U2OS xenografts with POLRMT shRNA virus injection. Together, POLRMT is overexpressed in human OS, promoting cell growth in vitro and in vivo. POLRMT could be a novel therapeutic target for OS.

## Introduction

Osteosarcoma (OS) is the most common bone malignancy among children and adolescents [1]. OS comprises 2.4% of all malignancies in pediatric patients [2–4]. It arises from the primitive transformed cells of mesenchymal origin [5, 6]. Under the current treatments, including systemic chemotherapy, radiation and local control surgery, the 5-year overall survival of OS is close to 70% [1]. For patients with metastatic and recurrent OS, the prognosis is often poor [5, 6]. One possibility is that OS cells are commonly resistant to chemotherapeutic agents [5, 6]. In recent years, molecularly-targeted therapies have drawn significant attentions for OS, and several of these agents are currently under different stages of clinical studies [6–10].

RNA polymerase mitochondrial (POLRMT) is a key component of mitochondrial transcription machinery [11, 12]. POLRMT and two other mitochondrial proteins, mitochondrial transcription factor A (TFAM) and transcription factor B2 (TFB2M), are essential for initiation and progression of promoter-specific mitochondrial DNA (mtDNA) transcription [11, 12]. Furthermore, the synthesis of RNA primers is also dependent on POLRMT, which is required for replication of mtDNA [13, 14].

Recent studies have proposed a cancer-promoting activity by POLRMT. In acute myeloid leukemia (AML) cells shRNA-induced knockdown of POLRMT reduced oxidative phosphorylation (OXPHOS), cell growth and viability [15]. Furthermore, 2-C-methyladenosine, a POLRMT inhibitor, exerted potent anti-tumor response in AML cells [15]. POLRMT and mitochondrial biogenesis levels are significantly elavated in breast cancer cells, important for cancer cell growth, and autophagy resistance [16, 17]. Bonekamp et al., have recently developed the first-in-class specific POLRMT inhibitors to block mitochondrial transcription [18]. These POLRMT inhibitors potently suppressed cancer cell growth in mice [18]. In the present study we showed that POLRMT is overexpressed in human OS, promoting OS cell growth in vitro and in vivo.

## Results

### POLRMT overexpression in human OS

**Fig. 1:**
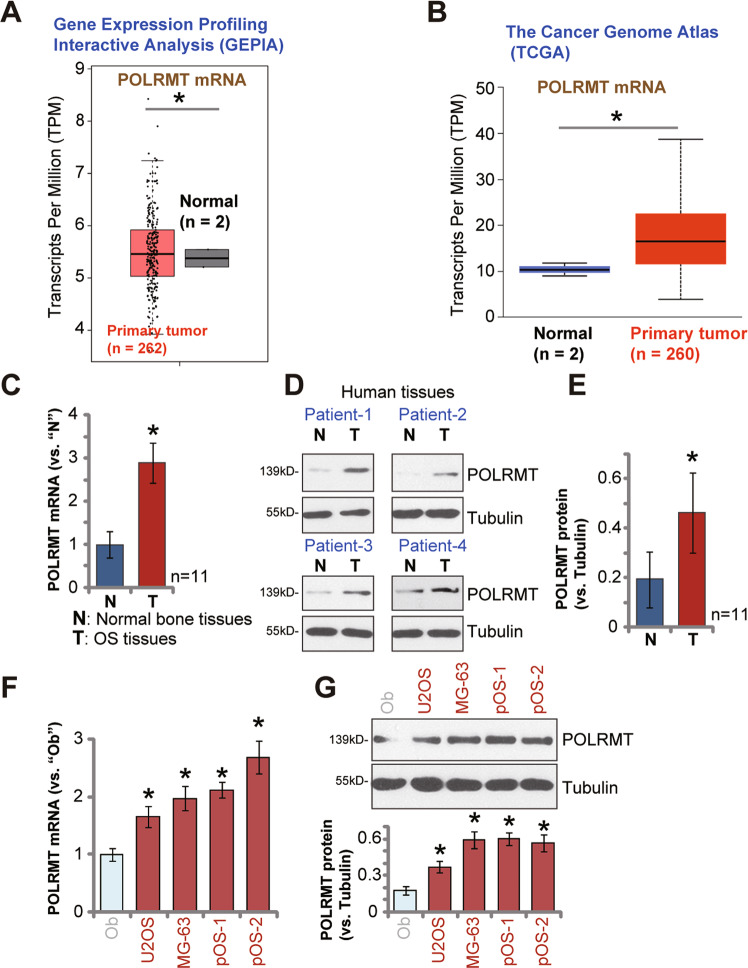
POLRMT overexpression in human OS. GEPIA database (**A**) and TCGA cohorts (**B**) showed the relative *POLRMT* mRNA expression in listed osteosarcoma (OS) tumor tissues and normal tissues. Expression of *POLRMT* mRNA and protein in OS tumor tissues (“T”) and the surrounding normal bone tissues (“N”) of eleven (11) OS patients was shown, with results quantified (**C**–**E**). Expression of *POLRMT* mRNA and protein in established OS cell lines (U2OS and MG63), primary human OS cells (pOS-1 and pOS-2, derived two patients), as well as in primary human osteoblasts (“Ob”) was shown, with results quantified (**F**, **G**). Data were presented as mean ± standard deviation (SD). *P < 0.05 versus normal tissues/“Ob” cells.

Gene Expression Profiling Interactive Analysis (GEPIA) database was consulted to examine *POLRMT* mRNA expression profile in human OS. GEPIA parameters were set as follows: (|Log2FC| Cutoff: 1; *P* value Cutoff:0.05). Results showed that *POLRMT* mRNA transcripts in OS tumor tissues (*n* = 262) were significantly higher than those in normal bone tissues (*n* = 2) (Fig. 1A). We also downloaded the raw mRNA expression data of sarcoma from TCGA database (https://portal.gdc.cancer.gov/). A total of 264 samples were collected, including two normal samples and 262 sarcoma samples. Results showed that *POLRMT* mRNA upregulation in OS tumor tissues (*P* < 0.05 versus normal bone tissues) (Fig. 1B).

To confirm the bioinformatics observation, we examined POLRMT expression in local human OS tissues. OS tumor tissues (“T”) and surrounding normal bone tissues (“N”) were derived from a total of eleven (*n* = 11) primary OS patients [19]. qRT-PCR assay results, Fig. 1C, demonstrated that *POLRMT* mRNA levels in OS tumor tissues were significantly higher than those in normal bone tissues (*P* < 0.05, Fig. 1C). Analyzing POLRMT protein expression, by Western blotting assays, showed that POLRMT protein levels were elevated as well in OS tissues (Fig. 1D, E). POLRMT blotting data of four represent patients, Patient-1 to Patient-4, were presented (Fig. 1D). Quantification analyses integrating Western blotting data from all 11 sets of human tissues were presented as well (Fig. 1E).

We also tested POLRMT expression in human OS cells. As shown, *POLRMT* mRNA (Fig. 1F) and protein (Fig. 1G) levels in established OS cell lines (U2OS and MG63) and primary human OS cells (pOS-1 and pOS-2, derived from two different patients [19]) were significantly higher than those in the primary human osteoblasts (“Ob”) (Fig. 1F, G). These results show that POLRMT is overexpressed in human OS tissues and different human OS cells.

### POLRMT silencing produces significant anti-cancer activity in OS cells

**Fig. 2:**
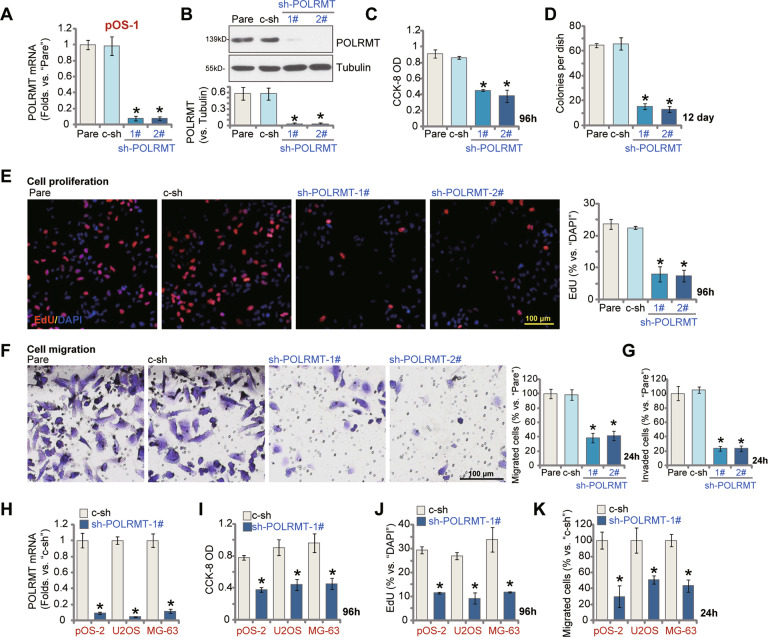
POLRMT silencing produces significant anti-cancer activity in OS cells. The primary human OS cells (pOS-1 and pOS-2, derived from two different patients) or established OS cell lines (U2OS and MG63), were cultured in polybrene-containing medium and infected with POLRMT shRNA lentivirus (sh-POLRMT-1#/sh-POLRMT-2#) or scramble control shRNA lentivirus (c-sh); Stable cells were established after puromycin selection. Expression of *POLRMT* mRNA and protein was shown (**A**, **B**, **H**); Cells were further cultured for applied time periods, cell viability (CCK-8 OD, **C**, **I**), colony formation (**D**) and proliferation (by recording nuclear EdU ratios, **E**, **J**) as well as cell migration and invasion (“Transwell” assays, **F**, **G**, **K**) were tested by the described methods, with results quantified. Expression of listed proteins was quantified and normalized to the loading control (**B**). “Pare” stands for the parental control cells. Error bars stand for mean ± standard deviation (SD, *n* = 5). *P < 0.05 versus “Pare” cells or “c-sh” cells. Experiments in this figure were repeated five times. Scale bar = 100 μm (**E**, **F**).

Next shRNA strategy was employed to silence POLRMT in OS cells. As described, two lentiviral shRNAs, targeting non-overlapping sequences of POLRMT, sh-POLRMT-1# and sh-POLRMT-2#, were separately transduced to pOS-1 primary cancer cells. Following selection by puromycin, stable cells were established. Analyzing *POLRMT* mRNA expression, using qRT-PCR assays, confirmed that each of the applied shRNA resulted in over 95% knockdown of *POLRMT* mRNA in pOS-1 cells (Fig. 2A). POLRMT protein levels were dramatically reduced as well in POLRMT shRNA-expressing pOS-1 cells (Fig. 2B). Testing cell viability, using CCK-8 assays, demonstrated that POLRMT shRNA significantly decreased the viability of pOS-1 cells (Fig. 2C). Moreover, clonogenicity assay results, Fig. 2D, showed that the number of viable pOS-1 cell colonies was largely decreased after POLRMT silencing. Evidenced by decreased EdU-positive nuclei ratio (Fig. 2E), we demonstrated that POLRMT shRNA inhibited pOS-1 cell proliferation. Further experiment results showed that POLRMT silencing potently suppressed pOS-1 cell migration and invasion, which were tested by “Transwell” (Fig. 2F) and “Matrigel Transwell” (Fig. 2G) assays, respectively. As expected, the scramble control shRNA, c-sh, failed to significantly alter POLRMT expression (Fig. 2A, B) and pOS-1 cell behaviors (Fig. 2C–G).

In addition, the sh-POLRMT-1# lentivirus was added to another primary human OS cells (pOS-2) and established OS cell lines (U2OS and MG63). Stable cells were again established after puromycin selection. Results from qRT-PCR assays demonstrated that sh-POLRMT-1# resulted in 80–90% silencing of *POLRMT* mRNA in the primary and established OS cells (Fig. 2H). In these OS cells, POLRMT shRNA potently inhibited cell viability, proliferation and migration, tested by CCK-8 (Fig. 2I), nuclear EdU staining (Fig. 2J) and “Transwell” (Fig. 2K) assays, respectively. These results showed that POLRMT shRNA inhibited OS cell viability, proliferation, migration, and invasion.

### POLRMT silencing provokes apoptosis in OS cells

**Fig. 3:**
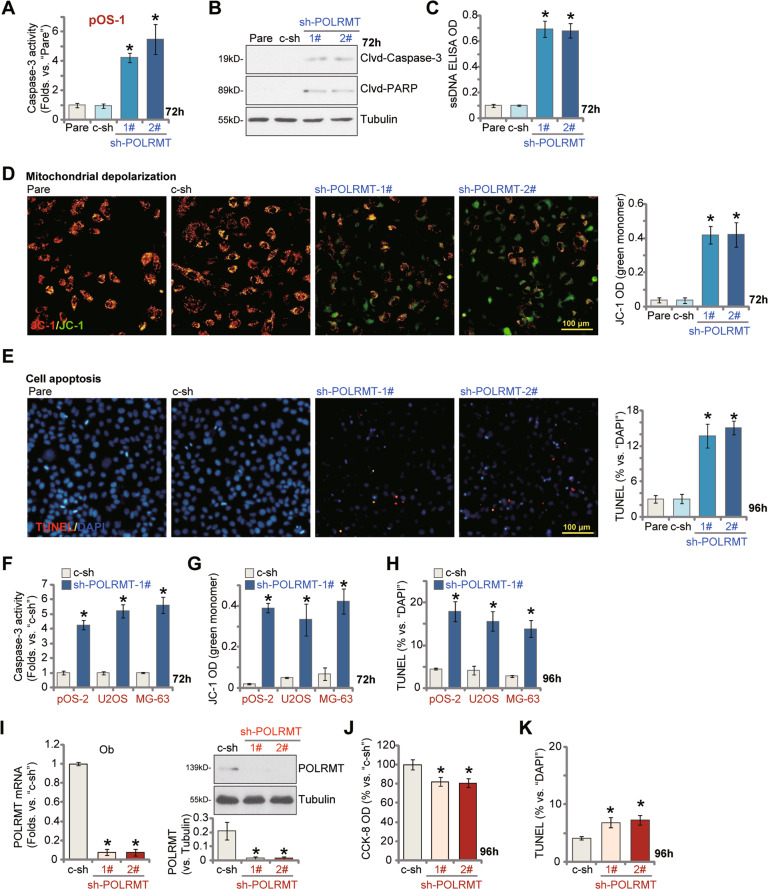
POLRMT silencing provokes apoptosis in OS cells. The primary human OS cells (pOS-1 and pOS-2, derived from two different patients) or established OS cell lines (U2OS and MG63), were cultured in polybrene-containing medium and then infected with applied POLRMT shRNA lentivirus (sh-POLRMT-1#/sh-POLRMT-2#) or scramble control shRNA lentivirus (c-sh); Stable cells were established after puromycin selection; Cells were further cultured for applied time periods, the relative caspase-3 activity (**A**, **F**), caspase-3-PARP cleavages (**B**), single strand DNA (ssDNA) contents (ELISA assays, **C**) and mitochondrial depolarization (by testing JC-1 green monomer intensity, **D**, **G**) were tested by the listed assays; Cell apoptosis was tested by recording nuclear TUNEL ratio (**E**, **H**). The primary human osteoblasts (“Ob”) were cultured in polybrene-containing medium and then infected with applied POLRMT shRNA lentivirus (sh-POLRMT-1#/sh-POLRMT-2#) or scramble control shRNA lentivirus (c-sh); Stable cells were established after puromycin selection; Expression of *POLRMT* mRNA and listed proteins was shown (**I**); Cells were further cultured for 96 h, with cell viability and apoptosis tested by CCK-8 (**J**) and the nuclear TUNEL staining (**K**) assays, respectively. “Pare” stands for the parental control cells. Error bars stand for mean ± standard deviation (SD, *n* = 5). *P < 0.05 versus “Pare” cells or “c-sh” cells. Experiments in this figure were repeated five times. Scale bar = 100 μm (**D**, **E**).

Experiments were performed to test whether POLRMT silencing could provoke apoptosis activation in OS cells. As shown in Fig. 3A, the relative caspase-3 activity was significantly increased in POLRMT shRNA-expressing pOS-1 primary cancer cells. Moreover, cleavages of caspase-3 and its substrate PARP (poly ADP-ribose polymerase) were significantly increased in POLRMT-silenced pOS-1 cells (Fig. 3B). Indicating DNA damage, we found that single strand DNA (ssDNA) contents were significantly increased in stable pOS-1 cells with POLRMT shRNA (Fig. 3C). In addition, JC-1 green monomer accumulation was detected in pOS-1 cells expressing the POLRMT shRNA (Fig. 3D), indicating mitochondrial depolarization [20, 21]. To confirm apoptosis activation we found that TUNEL-positive nuclei ratio was significantly increased in POLRMT-silenced pOS-1 cells (Fig. 3E). These results confirmed that POLRMT silencing by targeted shRNA provoked caspase-apoptosis activation in pOS-1 cells. The scramble control shRNA (c-sh), unsurprisingly, failed to induce caspase activation and apoptosis in pOS-1 cells (Fig. 3A–E).

In the primary pOS-2 cells and OS cell lines (U2OS and MG63), POLRMT silencing by shRNA (sh-POLRMT-1#) induced similar results, causing caspase-3 activation (Fig. 3F) and mitochondrial depolarization (JC-1 green monomer accumulation, Fig. 3G). Significant apoptosis activation, evidenced by increased TUNEL-positive nuclei ratio, was detected as well in the OS cells expressing POLRMT shRNA (Fig. 3H). Therefore, POLRMT silencing provoked apoptosis activation in OS cells.

In the primary human osteoblasts (“Ob”), stable transfection of the lentiviral POLRMT shRNA (sh-POLRMT-1#/-2#) resulted in robust silencing of *POLRMT* mRNA and protein (Fig. 3I). Significantly, POLRMT shRNA only resulted in minor but significant viability (CCK-8 OD) reduction (Fig. 3J) and apoptosis activation (tested by the TUNEL-positive nuclei ratio increase, Fig. 3K) in the human osteoblasts.

### CRISPR/Cas9-induced POLRMT knockout produces significant anti-cancer activity in OS cells

**Fig. 4:**
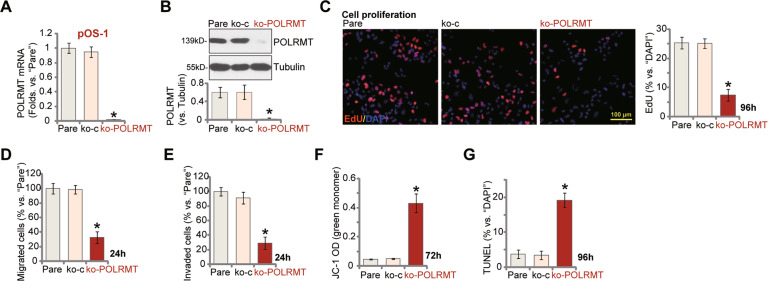
CRISPR/Cas9-induced POLRMT knockout produces significant anti-cancer activity in OS cells. The Cas9-expressing stable pOS-1 cells were further transfected with the lenti-POLRMT-sgRNA construct (ko-POLRMT) or the empty vector (“koC”), stable cells were established. Expression of *POLRMT* mRNA and protein was shown (**A**, **B**); Cells were further cultured for applied time periods, cell proliferation (nuclear EdU staining assays, **C**), migrated and invaded cell number (**D**, **E**), as well as mitochondrial depolarization (by testing JC-1 green monomer intensity, **F**) and cell apoptosis (by recording TUNEL-positive nuclei ratio, **G**) were tested. “Pare” stands for the parental control cells. Error bars stand for mean ± standard deviation (SD, *n* = 5). *P < 0.05 versus “Pare” cells. Experiments in this figure were repeated five times. Scale bar = 100 μm (**C**).

To further support the function of POLRMT in OS cells, we next utilized CRISPR/Cas9 strategy to KO POLRMT in OS cells. As described, the Cas9-expressing stable pOS-1 cells were further transfected with lenti-POLRMT-sgRNA construct. Following puromycin selection and POLRMT KO screening, the single stable POLRMT KO pOS-1 cells were established: ko-POLRMT cells. qRT-PCR and Western blotting assays were performed to examine POLRMT expression, showing complete POLRMT KO in ko-POLRMT cells (Fig. 4A, B). In pOS-1 cells CRISPR/Cas9-induced POLRMT KO potently inhibited cell proliferation (decreased EdU-positive nuclei ratio, Fig. 4C), migration (results quantified in Fig. 4D) and invasion(Fig. 4E). Moreover, mitochondrial depolarization (tested by JC-1 green monomer intensity increase, Fig. 4F) and apoptosis activation (tested by the TUNEL-positive nuclei ratio increase, Fig. 4G) were detected in ko-POLRMT pOS-1 cells. As expected, the control lenti-sgRNA construct, koC, failed to significantly alter POLRMT expression (Fig. 4A, B) and pOS-1 cell function (Fig. 4C–G).

### Ectopic overexpression of POLRMT accelerates OS cell proliferation, migration, and invasion

**Fig. 5:**
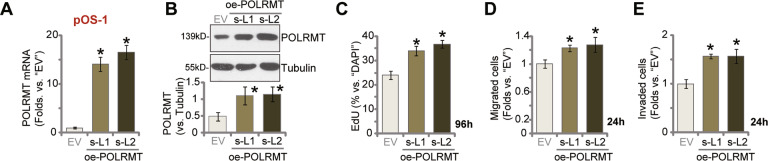
Ectopic overexpression of POLRMT accelerates OS cell proliferation, migration and invasion. POLRMT-overexpressed pOS-1 cells (oe-POLRMT-sL1 and oe-POLRMT-sL2, two stable cell lines) or the control pOS-1 cells with empty vector (EV) were established; *POLRMT* mRNA and protein were tested (**A**, **B**); Cells were further cultured for applied time periods, cell proliferation (nuclear EdU staining assays, **C**), migration and invasion (**D**, **E**, “Transwell” assays) were tested, with results quantified. Error bars stand for mean ± standard deviation (SD, *n* = 5). *P < 0.05 versus “EV” cells. Experiments in this figure were repeated five times.

Since POLRMT silencing or KO induced robust anti-OS cell activity, we proposed that ectopic overexpression of POLRMT might exert opposite function. Therefore, a lentiviral construct encoding full-length POLRMT cDNA was stably transduced to pOS-1 cells. Via selection by puromycin two stable cell lines with PLORMT overexpression, oe-POLRMT-sL1 and oe-POLRMT-sL2, were established. Testing POLRMT expression, via qRT-PCR and Western blotting assays, showed that *PLORMT* mRNA (Fig. 5A) and protein (Fig. 5B) levels were significantly increased in oe-POLRMT pOS-1 cells (*P* < 0.05 versus control cells with empty vector/“EV”). Ectopic overexpression of POLRMT accelerated OS cell proliferation, as the EdU-positive nuclei ratio was significantly increased in oe-POLRMT-sL1 cells and oe-POLRMT-sL2 cells (Fig. 5C). Moreover, POLRMT overexpression in pOS-1 cells augmented cell migration and invasion, tested by “Transwell” (Fig. 5D) and “Matrigel Transwell” (Fig. 5E) assays, respectively.

### In OS cells mtDNA contents, mitochondrial transcripts and expression respiratory chain complex subunits are decreased after POLRMT depletion

**Fig. 6:**
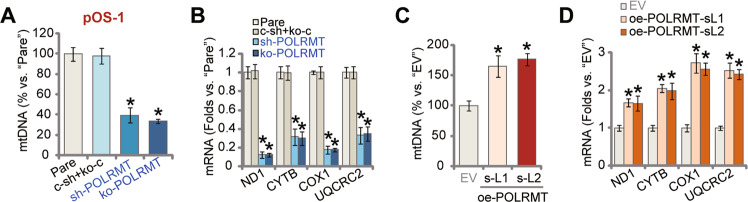
In OS cells mtDNA contents, mitochondrial transcripts and expression respiratory chain complex subunits are decreased after POLRMT depletion. Stable pOS-1 cells with sh-POLRMT-1# (“sh-POLRMT”), the lenti-POLRMT-sgRNA construct (“ko-POLRMT”) or scramble control shRNA plus the empty vector (“c-sh+koC”), as well as POLRMT-overexpressed pOS-1 cells (oe-POLRMT-sL1 and oe-POLRMT-sL2, two stable cell lines) or the control cells with empty vector (EV), were established; Levels of mtDNA (**A**, **C**) and listed mRNAs (qRT-PCR assays, **B**, **D**) were examined. “Pare” stands for parental control cells. Data were presented as mean ± standard deviation (SD, *n* = 5). *P < 0.05 versus “Pare”/“EV” cells. The experiments were repeated five times with similar results obtained.

Studies have implied that POLRMT silencing or inhibition would result in decreases in mtDNA copies and mitochondrial transcripts [18]. As shown, in pOS-1 cells with POLRMT shRNA (sh-POLRMT-1#, see Figs. 2 and 3) or the lenti-POLRMT-sgRNA construct (ko-POLRMT, see Fig. 4), mtDNA contents were significantly decreased (*P* < 0.05 versus parental control cells, Fig. 6A). Levels of mitochondrial transcripts, *ND1* and *CYTB*, were decreased as well after POLRMT silencing or KO (Fig. 6B). Moreover, mRNA levels of respiratory chain complex subunits, *NDUFB8* and *UQCRC2* [18], were reduced in sh-POLRMT and ko-POLRMT pOS-1 cells (Fig. 6B). Conversely, mtDNA contents (Fig. 6C) as well as mRNA levels of *ND1*, *CYTB*, *NDUFB8* and *UQCRC2* (Fig. 6D) were significantly increased in POLRMT-overexpressed pOS-1 cells: oe-POLRMT-sL1 and oe-POLRMT-sL1 (*P* < 0.05 versus “EV” control cells).

### POLRMT silencing inhibits U2OS xenograft growth in nude mice

**Fig. 7:**
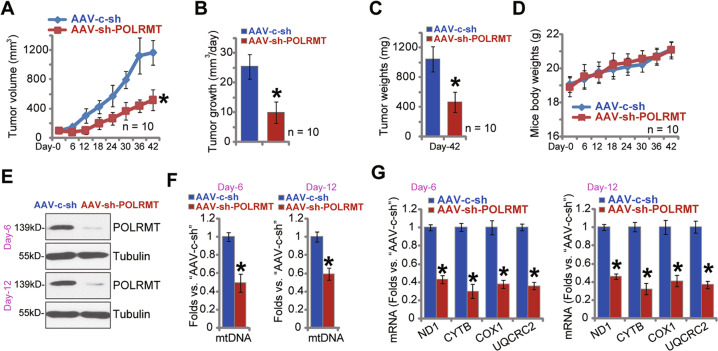
POLRMT silencing inhibits U2OS xenograft growth in nude mice. U2OS xenograft-bearing nude mice were randomly assigned into two groups (ten mice per group, *n* = 10), subject to intratumoral injection of AAV-POLRMT shRNA (“AAV-sh-POLRMT”) or AAV scramble control shRNA (“AAV-c-sh”). Viruses were injected daily for 6 consecutive days. Tumor volumes (**A**) and mice body weights (**D**) were recorded every 6 days (“Day-0 to Day-42”); Estimated daily tumor growth was calculated using the described formula (**B**). At Day-42 U2OS xenografts were isolated and individually weighted (**C**). At Day-6 and Day-12, one xenograft of each group was isolated (total four xenografts). Western blotting (**E**) and qRT-PCR (**G**) assays were employed to examine listed genes in fresh tumor tissue lysates, with mtDNA contents tested as well (**F**). Data were presented as mean ± standard deviation (SD). *P < 0.05 versus “AAV-c-sh” group.

We tested the potential effect of POLRMT on OS cell growth in vivo. As described, U2OS cells, at 5 × 10^6^ cells per mouse, were *s.c*. injected to the flanks of nude mice. Within 25 days, U2OS xenograft tumors were established with the volume close to 100 mm^3^ (“Day-0”). At 10 mice per group (*n* = 10), U2OS xenograft-bearing nude mice were randomly assigned into two groups. The treatment group received intratumoral injection of AAV-POLRMT shRNA (“AAV-sh-POLRMT”), daily for 6 consecutive days. The control group mice, on the other hand, received intratumoral injection of AAV scramble control shRNA (“AAV-c-sh”). In Fig. 7A, the tumor growth curve results showed that the growth of U2OS xenografts with AAV-sh-POLRMT injection was significantly slower than control xenografts with AAV-c-sh injection. The estimated daily tumor growth was calculated using the following formula: (Tumor volume at Day-42—Tumor volume at Day-0)/42. Results, Fig. 7B, demonstrated that intratumoral injection of AAV-sh-POLRMT potently inhibited U2OS xenograft growth in mice. At experimental Day-42, all xenograft tumors were carefully separated and weighted individually. As shown, the AAV-sh-POLRMT-injected U2OS xenografts were significantly lighter than control xenografts with AAV-c-sh injection (Fig. 7C). The mice body weights, shown in Fig. 7D, were not significantly different between the two groups. These results showed that POLRMT silencing inhibited U2OS xenograft growth in nude mice.

At Day-6 and Day-12, one U2OS xenograft tumor from each group was isolated. The fresh tissue lysates from the four tumors were achieved and analyzed. Western blotting assay results, Fig. 7E, confirmed that POLRMT protein expression was significantly downregulated in AAV-sh-POLRMT-injected U2OS xenografts. In addition, mtDNA contents were decreased (Fig. 7F). Moreover, the mitochondrial transcripts (*ND1* and *CYTB*) as well as mRNA expression of respiratory chain complex components (*NDUFB8* and *UQCRC2*) were inhibited in U2OS xenografts with AAV-sh-POLRMT injection (Fig. 7G). Therefore, in line with the in vitro findings, POLRMT silencing decreased mtDNA contents, mitochondrial transcripts and expression of respiratory chain complex components in U2OS xenografts.

## Discussion

OXPHOS generates ATP by transport of electrons to electron transport chain, the latter is composed of a number of transmembrane protein complexes in the mitochondrial inner membrane [22, 23]. Recent studies have implied that OXPHOS levels are upregulated in a number of different cancers, including leukemia, lymphomas, breast cancer, pancreatic cancer, certain melanomas, and endometrial carcinoma [22, 23]. OXPHOS inhibitors could therefore be utilized to target these cancers, showing promising anti-cancer efficiency in preclinical studies [22, 23]. Recent studies have implied that mtDNA is required for tumorigenesis and progression for cancer cells, mediating resistance to cytotoxic drugs [24]. Cancer cell growth could be largely diminished with mtDNA depletion [24, 25]. mtDNA transcription arrest could therefore result in significant anti-tumor activity [26–28].

POLRMT is essential for mtDNA transcription and OXPHOS [26–28], represents as a novel therapeutic target for human cancer [15, 18, 29]. POLRMT overexpression in AML is associated increased mtDNA copy number and lower overall survival [29]. In AML cells, POLRMT knockdown by shRNA reduced mitochondrial gene expression and inhibited OXPHOS and expression of respiratory chain complex subunits, causing significant cell death [15]. 2-C-methyladenosine (2-CM), a mitochondrial transcription inhibitor, resulted in significant cancer cell death [15]. A recent study by Bonekamp and colleagues developed a first-in-class POLRMT direct inhibitor, inhibitor of mitochondrial transcription 1 (IMT1). POLRMT inhibition by IMT1 induced robust cancer growth arrest [18]. Therefore, POLRMT inhibition or silencing shall impair mtDNA transcription and OXPHOS, resulting in significant cancer growth inhibition and cancer cell death [15, 18, 29].

Our results indicated that POLRMT could be an important therapeutic target of OS. GEPIA and TCGA database show that POLRMT transcripts are significantly elevated in human OS. Furthermore, *POLRMT* mRNA and protein are upregulated in local OS tissues as well as in established and primary human OS cells. But low expression is detected normal bone tissues and human osteoblasts. Oran et al., have also reported that POLRMT expression is elevated in U2OS cells, where it is a direct transcriptional target of the MYC oncoproteins [30].

In primary human OS cells and established cell lines, shRNA-induced knockdown of POLRMT largely inhibited cell viability, proliferation, migration and invasion, and inducing apoptosis activation. POLRMT KO, using CRISPR/Cas9 gene editing method, similarly induced profound anti-OS cell activity. Conversely, forced overexpression of POLRMT in primary OS cells accelerated cell proliferation, migration and invasion. In vivo, intratumoral injection of POLRMT shRNA AAV potently inhibited U2OS xenograft growth in nude mice. Importantly, the toxicity of the silencing for POLRMT was minor in the primary human osteoblasts as compared to that observed in OS cells. These results, together with results from other cancer studies [15, 16, 18, 31], strongly supported the pro-tumorigenic activity of POLRMT overexpression in human cancer. Targeting POLRMT could be a novel strategy to inhibit OS progression.

The mitochondrial functions are essential in tumorigenesis of OS [32, 33]. Bonuccelli et al. have found that OXPHOS and ATP-production were both significantly increased in OS cells after co-culture with mesenchymal stem cells, resulting in more aggressive behaviors of tumor cells, in particular enhanced cell migration [34]. Day et al., suggested that Bcl-xL can exert an anti-apoptotic effect by stimulating OXPHOS in OS cells [35]. Here we found that mtDNA contents, mitochondrial transcripts and expression of respiratory chain complex subunits were significantly reduced in POLRMT-silenced OS cells and U2OS xenografts, but were increased after POLRMT overexpression. Therefore, POLRMT-driven OS cell progression could be due to its effect in promoting OXPHOS. The underlying signaling mechanisms may warrant further characterizations.

## Conclusion

Our results showed that POLRMT is overexpressed in human OS, promoting OS cell growth in vitro and in vivo. POLRMT could be a novel therapeutic target for better OS therapy.

## Material and methods

### Ethics

All the methods applied in this study were carried out according to the ethics guidelines by all authors’ institutions.

### Chemicals, reagents, and antibodies

Puromycin, polybrene, fetal bovine serum (FBS), medium, antibiotics and other cell culture reagents were purchased from Sigma (St. Louis, Mo). Cell counting kit -8 (CCK-8) was provided by Dojindo (Kumamoto, Japan). The fluorescence dyes, including EdU (5-Ethynyl-2′-deoxyuridine), DAPI (4′,6-diamidino-2-phenylindole), TUNEL (Terminal deoxynucleotidyl transferase dUTP nick end labeling) and JC-1, were provided by Thermo-Fisher Invitrogen (Shanghai, China). The anti-POLRMT antibody was purchased from Abcam (ab228576, Shanghai, China). The cleaved caspase antibody sampler Kit (#9929) and anti-β-Tubulin (#2146) were provided by Cell Signaling Technologies (Beverly, MA).

### Human tissues

OS tumor tissues and matched surrounding normal bone tissues were from a total of eleven (11) primary OS patients (provided by Dr. Cao’s group at Soochow University [19, 36]) with written-informed consents. All patients were administrated at authors institutions, received no prior therapies before surgeries. Fresh tissues were stored in liquid nitrogen. The protocols of using human tissues were approved by the Ethics Committee of The First Affiliated Hospital of Zhengzhou University, in according to the principles of Declaration of Helsinki.

### OS cell culture

U2OS and MG-63 established human OS cell lines were from Dr. Zhang at Medical College of Southeast University [37–39]. The primary human OS cells, derived from two written-informed consent OS patients, were from Dr. Cao at Soochow University [19, 36]. The primary human osteoblasts were from Dr. Ji at Nanjing Medical University [40, 41]. The detailed protocols of culturing established and primary human OS cells as well as human osteoblasts were described early [37–41]. Mycoplasma-microbial contamination examination, STR profiling, population doubling time and morphology were checked to confirm the genotypes. The written-informed consent was obtained from each participant. The protocols of using primary human cells were approved by the Ethics Committee of The First Affiliated Hospital of Zhengzhou University, in according to the principles of Declaration of Helsinki.

### Cell viability

In brief, OS cells with applied genetic modifications were seeded into 96-well plates (at 3 × 10^3^ cells per well) and cultured for 96 h. Cell viability was examined by CCK-8 kits. In each well CCK-8 optical density (OD) at 490 nm was recorded.

### Clonogenicity assay

OS cells with applied genetic modifications were re-suspended in complete medium with 0.5% agar (Sigma). Cell suspensions (3 × 10^4^ cells in each plate) were thereafter added on top of the 100 mm-diameter culture dishes. The medium was renewed every 2 days. After 12 days, viable large colonies were stained and manually counted.

### Western blotting

Cells or tissues were incubated with cell lysis buffer as described [42, 43]. The achieved protein lysates (30 μg of each treatment) were separated by 10–12% SDS-PAGE and thereafter transferred to polyvinylidene fluoride blots (Millipore, Invitrogen, Shanghai, China). Afterwards, blots were blocked and incubated with applied primary and secondary antibodies. Signal detection and data quantification were described in detail in elsewhere [42, 43].

### Quantitative reverse transcription PCR (qRT-PCR)

Total RNA was extracted via TRIzol reagents (Invitrogen; Thermo Fisher Scientific). Complementary DNA (cDNA) was then synthesized through a PrimeScript RT Reagent kit (Takara, Shanghai, China). qRT-PCR assays were carried out by an ABI 7900 Fast Real-Time PCR system (Applied Bioscience; Thermo Fisher Scientific.) through a Power SYBR Green PCR Master mix kit (Applied Biosystems, Shanghai, China). A 2^−ΔΔCt^ method was utilized for data quantification. *Glyceraldehyde-3-phosphate dehydrogenase* (*GAPDH*) was always examined as internal control. mRNA primers were provided by Dr. Shi’s group at Soochow University [31].

### TUNEL staining

U2OS cells with applied genetic treatments were seeded into 12-well plates (5 × 10^4^ cells per well) and cultured for 96 h. A TUNEL In Situ Cell Apoptosis Detection Kit (Invitrogen) was employed. Briefly, TUNEL and DAPI fluorescence dyes were added to OS cells, visualized under a fluorescent microscope (Leica). TUNEL ratio (% versus DAPI) was calculated from at least 1500 cell nuclei in five random views (1 × 200) in each condition [38].

### Caspase-3 activity assay

U2OS cells with applied genetic treatments were seeded into six-well tissue plates at 1.2 × 10^5^ cells per well. Twenty μg of cytosolic protein extracts were added to the attached caspase assay buffer [37, 38] along with the caspase-3 substrate Ac-DEVD-AFC. Using a spectrofluorometer the released AFC intensity was test at 450 nm.

### EdU staining

OS cells with applied genetic treatments were seeded into 12-well plates (at 5 × 10^4^ cells per well) for 96 h. To test cell proliferation, an EdU Apollo-567 Kit (Invitrogen) was employed. Briefly, EdU and DAPI fluorescence dyes were added to OS cells, visualized under a fluorescent microscope (Leica). For each condition, at least 1500 cell nuclei from five random views (1 × 200) were included to calculate EdU-positive nuclei ratio (% versus DAPI).

### Transwell assays

“Transwell” chambers with12 μm pore (Corning, Shanghai, China) were utilized for in vitro cell migration and invasion assays. OS cells with applied genetic modifications were placed on the upper chambers (at 2 × 10^4^ cells per chamber, in 200 µL DMEM). The lower chambers were filled with complete medium with 10% FBS. Following 24 h, the non-migrated OS cells on the upper surface were removed carefully. On the lower surface migrated cells were fixed and stained (with crystal violet). For invasion assays, the chambers were coated with Matrigel (BD Biosciences, Shanghai, China).

### Mitochondrial depolarization

OS cells with applied genetic treatments were seeded into 12-well plates (at 3 × 10^4^ cells per well) for 72 h. JC-1 dye was then added for 2 h. JC-1 green monomer intensity, reflecting mitochondrial depolarization, was detected at the test-wavelength of 490 nm. JC-1 images, integrating both green (490 nm) and red (625 nm) fluorescence channels, were presented.

### POLRMT silencing or overexpression

GV369 lentiviral constructs encoding shRNAs against non-overlapping sequences of POLRMT (sh-POLRMT-1# and sh-POLRMT-2#), as well as the GV369 lentiviral construct encoding full-length *POLRMT* cDNA were provided by Dr. Shi at Soochow University [31]. Each was transduced to HEK-293T cells together with the lentivirus helper plasmids. Within 48 h lentivirus was generated, enriched and filtered. OS cells were seeded into six-well plates (at 1 × 10^5^ cells per well), cultured in polybrene-containing complete medium and infected with the lentivirus (MOI = 10). After 24 h, cells were cultured in fresh complete medium. Puromycin was added to select stable cells. POLRMT expression in stable OS cells was verified by qRT-PCR and Western blotting assays.

### POLRMT knockout (KO)

OS cells were initially seeded into six-well plates (at 1 × 10^5^ cells per well), cultured in polybrene-containing complete medium and transfected with a LentiCas9-puro construct (Genechem). Stable Cas9 cells were established after puromycin selection. Cells were further transfected with Lenti-POLRMT-sgRNA-puro construct (Genechem) for 24 h. Afterwards, cells were cultured in fresh complete medium. Puromycin was added to select stable cells. Stable cells were distributed into 96-well plates and subjected to POLRMT KO screening. POLRMT expression in the single stable OS cells was verified by qRT-PCR and Western blotting assays.

### mtDNA contents

Total DNA in OS cells was extracted, purified and quantified. For each treatment 7.5 ng/μl DNA was analyzed by quantitative PCR via a Taqman 2× Universal PCR mastermix (Applied Biosystems, Shanghai, China) and commercially available Taqman assay probes for *COX1* mtDNA (18 S). mtDNA contents in genetic modified OS cells were normalized to those in control OS cells.

### Xenograft assay

The nude mice were purchased from the experimental animal center of Soochow University (Suzhou, China): Half male and hale female, 7-week old, 18.3–19.2 grams. Mice were maintained under the 12 h light/dark cycle in standard environmental conditions (22.2 ± 1.5 °C, 46–55% r humidity). U2OS cells, at 5 × 10^6^ cells per mouse, were dissolved in 100 μL of RPMI and Matrigel (1:1) and were subcutaneously (*s.c*.) injected to right flanks of nude mice. Within 25 days, U2OS xenograft-bearing nude mice were established and the volume of each tumor close to 100 mm^3^. Afterward, mice were randomly assigned into two groups, with ten mice per group. One group mice were intratumorally injected with POLRMT shRNA adeno-associated virus (“AAV-sh-POLRMT”) and the other group mice received AAV packed scramble control shRNA (“AAV-c-sh”). AAV was injected daily for 6 consecutive days. Tumor dimensions were measured and tumor volume calculated using the following formula: Volume (V) = length × width × height × 0.5236. The animal protocols were according to the regulations of the IACUC and with the approval of Zhengzhou University.

### Statistical studies

Data with normal distribution were presented as mean ± standard deviation (SD). Statistical differences between multiple groups were analyzed by one-way ANOVA with post hoc Bonferroni test (SPSS version 20.0, SPSS Co., Chicago, CA). The two-tailed unpaired *T*-test was applied (Excel 2007, Microsoft) for the comparison of two groups. *P* < 0.05 was considered statistically significant.

## Data Availability

The original contributions presented in the study are included in the paper. Further inquiries can be directed to the corresponding authors.
